# Anomalous High-Energy Waterfall-Like Electronic Structure in 5 *d* Transition Metal Oxide Sr_2_IrO_4_ with a Strong Spin-Orbit Coupling

**DOI:** 10.1038/srep13036

**Published:** 2015-08-12

**Authors:** Yan Liu, Li Yu, Xiaowen Jia, Jianzhou Zhao, Hongming Weng, Yingying Peng, Chaoyu Chen, Zhuojin Xie, Daixiang Mou, Junfeng He, Xu Liu, Ya Feng, Hemian Yi, Lin Zhao, Guodong Liu, Shaolong He, Xiaoli Dong, Jun Zhang, Zuyan Xu, Chuangtian Chen, Gang Cao, Xi Dai, Zhong Fang, X. J. Zhou

**Affiliations:** 1 Beijing National Laboratory for Condensed Matter Physics, Institute of Physics, Chinese Academy of Sciences, Beijing 100190, China; 2 Collaborative Innovation Center of Quantum Matter, Beijing, China; 3 Technical Institute of Physics and Chemistry, Chinese Academy of Sciences, Beijing 100190, China; 4 Department of Physics and Astronomy, University of Kentucky, Lexington, KY 40506.

## Abstract

The low energy electronic structure of Sr_2_IrO_4_ has been well studied and understood in terms of an effective J_*eff*_ = 1/2 Mott insulator model. However, little work has been done in studying its high energy electronic behaviors. Here we report a new observation of the anomalous high energy electronic structure in Sr_2_IrO_4_. By taking high-resolution angle-resolved photoemission measurements on Sr_2_IrO_4_ over a wide energy range, we have revealed for the first time that the high energy electronic structures show unusual nearly-vertical bands that extend over a large energy range. Such anomalous high energy behaviors resemble the high energy waterfall features observed in the cuprate superconductors. While strong electron correlation plays an important role in producing high energy waterfall features in the cuprate superconductors, the revelation of the high energy anomalies in Sr_2_IrO_4_, which exhibits strong spin-orbit coupling and a moderate electron correlation, points to an unknown and novel route in generating exotic electronic excitations.

The transition metal oxides exhibit rich exotic physical properties such as high temperature superconductivity and colossal magnetoresistance that have become a central theme of modern condensed matter physics^1^,^2^. The insulating ground state of the *3d* transition metal oxides can generally be understood by the strong on-site Coulomb repulsion U, relative to its bandwidth W (U ≫ W), as proposed in the Mott-Hubbard model^1^. An insulator-metal transition can occur when W ≥ U. In comparison, in the *5d* transition metal oxides, the electron correlation is expected to become less strong due to the more spatially extended *5d* orbitals and a metallic ground state is expected^3^,^4^. It is thus surprising when it was found that the prototypical *5d* compound Sr_2_IrO_4_ is an antiferromagnetic insulator below the Neel temperature T_*N*_ ~ 240 K^5^,^6^,^7^,^8^. One popular scenario for the novel insulating ground state of Sr_2_IrO_4_ is the J_*eff*_ = 1/2 Mott insulator model driven by spin-orbit coupling^7^,^9^. In this model, five *5d* electrons occupy the t_2*g*_ orbitals which are split into a fully-filled J_*eff*_ = 3/2 quartet band with lower energy and a half-filled doublet band with J_*eff*_ = 1/2 close to the Fermi level (E_*F*_) by strong spin-orbit coupling. Since the width of the J_*eff*_ = 1/2 band is narrow, even a moderate Coulomb repulsion U can open up a gap, giving rise to the so-called J_*eff*_ = 1/2 Mott insulating ground state^7^. A number of experimental results are consistent with this scenario^7^,^10^,^11^,^12^,^13^,^14^,^15^,^16^,^17^,^18^. Moreover, in addition to the J_*eff*_ = 1/2 Mott scenario^7^, the slater-type mechanism is also found to be important in the formation of the insulating ground state in Sr_2_IrO_4_^20^,^21^,^22^. A consensus that has been reached is Sr_2_IrO_4_ being a typical system where the Mott- and slater-type behaviors coexist. In addition, Sr_2_IrO_4_ has attracted much attention because it exhibits a number of similarities to the parent compound La_2_CuO_4_ of high temperature cuprate superconductors in the crystal structure, electronic structure, magnetic structure, and even possible high temperature superconductivity that is predicted in doped Sr_2_IrO_4_^23^,^24^.

Angle-resolved photoemission spectroscopy (ARPES) is a powerful tool to directly probe the low energy electronic structures of solid materials^25^. While the ARPES results on Sr_2_IrO_4_ and related compounds within a relatively narrow energy window agree with the J_*eff*_ = 1/2 Mott insulator model^7^,^13^,^14^,^15^,^18^, little work has been done in studying its high energy electronic behaviors. In this paper, we report the observation of unusual high energy bands in Sr_2_IrO_4_. Our comprehensive angle-resolved photoemission study over a wide energy window reveals for the first time nearly-vertical bands in Sr_2_IrO_4_. Such exotic bands cannot be understood in terms of the band structure calculations; they cannot be understood within the J_*eff*_ = 1/2 Mott insulator model either. The observed high energy anomaly resemble the unusual high energy waterfall bands discovered in the high temperature cuprate superconductors^26^,^27^,^28^,^29^,^30^,^31^,^32^,^33^,^34^,^35^. These observations point to the significant role of the strong spin-orbit coupling, together with a moderate electron correlation, in giving rise to new high energy excitations in the *5d* transition metal oxides.

Figure 1 shows the constant energy contours of Sr_2_IrO_4_ at different binding energies. No spectral weight is present at the Fermi level (not shown in Fig. 1), consistent with the insulating nature of Sr_2_IrO_4_^5^,^6^,^7^. At a binding energy of 0.2 eV, the spectral weight appears first as a circular spot around the X(*π*, 0) and its equivalent locations (Fig. 1a). Further increase of the binding energy to 0.4 eV results in the enlargement of the spot into a square-shape and the emergence of spectral weight near the Γ (0, 0) point (Fig. 1b). When the binding energy increases to 0.8 eV, the strong spectral weight near X points vanishes with a formation of a few disconnected patches around X, while the spectral weight near Γ exhibits a petal-like shape with four leaves (Fig. 1c). The measured constant energy contours at low binding energy (0 ~ 0.4 eV) are consistent with those reported before^7^,^14^. The constant energy contours at an intermediate binding energy (e.g., 0.4 eV) are also consistent with the band structure calculations (Fig. 1d) that include both the on-site Coulomb repulsion U and the spin-orbit coupling^7^. In terms of the spin-orbit-coupling-driven Mott insulator model^7^, the unoccupied states are mainly the J_*eff*_ = 1/2 state, while the occupied states are a mixture of the J_*eff*_ = 1/2 and 3/2 states. Due to the strong spin-orbit coupling, the topmost low energy valence state at X is more with J_*eff*_ = 1/2 character (*β* sheet near X in Fig. 1d), while the topmost low energy valence state at Γ is more with J_*eff*_ = 3/2 character (*α* sheet near Γ in Fig. 1d)^7^. The consistency of the low energy electronic structure with the previous reports and the band structure calculations lays a foundation for our following investigation of high binding energy electronic structure in Sr_2_IrO_4_.

At high binding energies, we find that the electronic structure of Sr_2_IrO_4_ is quite unusual. Figure 2 shows band structure along two high-symmetry momentum cuts covering a large energy range till ~6 eV: one cut is across Γ (Fig. 2a–d), the other is across X (Fig. 2e–h) (for more momentum cuts, see Fig. S1, S2 and S3 in Supplementary Materials). Here we show both the original data (Fig. 2a,e), and their corresponding momentum-(Fig. 2b,f) and energy-second-derivative (Fig. 2c,g) images. The second-derivative images help to highlight the band structure more clearly although many features are already clear in the original data. Since momentum-second derivative image may miss the flat horizontal bands while the energy-second derivative image may miss the vertical bands, the energy- and momentum-second-derivative images are complementary to each other to provide a full picture. As seen in Fig. 2, at low binding energy (0 ~ 1 eV), two prominent bands are observed labeled as *α*_0_ and *β*_0_ (Fig. 2c,g) that are consistent with the previous reports^7^,^14^. However, at higher binding energy, the electronic structure becomes quite unusual. First, the momentum-second derivative images and energy-second-derivative images give rather different band structures for both the Γ and X momentum cuts. Second, as seen in Fig. 2b, a clear vase-shaped band (labeled as *α*_1_ in Fig. 2b) and a vertical waterfall-like band (labeled as *α*_2_ in Fig. 2b) are observed around the Γ point. The vertical band structure is present even beyond 3 eV up to ~6 eV (Fig. 2b). Note that these features are not due to the artifact of the momentum second-derivative image because they are already clear in the original data (Fig. 2a). Such features can also be identified clearly in the momentum distribution curves (MDCs) where the peaks corresponding to *α*_1_ and *α*_2_ bands are marked (Fig. 2d). Similar behaviors are observed for the momentum cut across the X point (Fig. 2e) where nearly vertical band structures (labeled as *α*_3_ and *β*_1_ in Fig. 2f) are observed up to 4 eV, and another set of vertical bands are seen even up to 6.5 eV (Fig. 2f). We note that such a high energy band anomaly was not revealed before because the previous ARPES measurements cover a relatively small energy range (0 ~ 2 eV)^7^,^13^,^14^,^15^,^18^. In fact, upon careful examination, some indications of the high energy waterfall-like features appear to be present in a recent ARPES study on Sr_2_IrO_4_^18^ which are consistent with our results.

The unusual high energy electronic structure of Sr_2_IrO_4_ is present over a large momentum space. Figure 3 shows the detailed momentum evolution of the high energy electronic structure: one is near the Γ region (Fig. 3a–d) and the other near the X(0, *π*) region (Fig. 3e–h). While the energy-second-derivative images (Fig. 3d,h) show normal two bands (*α*_0_ and *β*_0_) in the covered energy range as already seen in Fig. 2, nearly vertical bands are observed in the momentum-second-derivative images (Fig. 3c,g) in both cases for different momentum cuts. Furthermore, the constant energy contours exhibit dramatic evolution with the binding energy (Fig. 3a,e). The spectral weight distribution around the Γ point (Fig. 3a) changes from a pocket centered at Γ at a binding energy of 0.4 eV, to butterfly-shaped at 0.6 eV and 0.8 eV, to big-X-shaped at 1.2 eV and to dumbbell-shaped at 2.0 eV and 2.4 eV. It is interesting to note that the spectral weight distribution shows discrete four strong spots at 0.6 eV and 0.8 eV, other than a continuous contour. From Fig. 3c,d, it becomes clear that the drastic spectral distribution change with the binding energy above 1.0 eV is directly related with the presence of the nearly-vertical *α*_1_ and *α*_2_ bands. It is also clear from Fig. 3b that, moving away from the cut across Γ (cut 1), the vase-shaped band and vertical structure persist for the cuts 2 and 3. The same is true for the X point constant energy contours (Fig. 3e) and the momentum-dependent band structures (Fig. 3f–h). First, the constant energy contours near X also exhibit an obvious evolution with the binding energy (Fig. 3e). Second, the vertical bands are present over a large area of momentum space near X (Fig. 3g).

Figure 4 summarizes the band structure of Sr_2_IrO_4_ measured along three typical high-symmetry momentum cuts (Fig. 4c–e). For comparison, the band structures of Sr_2_IrO_4_ in the antiferromagnetic state are also calculated using the DMFT method with U = 2.5, J = 0 and *β* = 100 (*β* = 1/k_*B*_T, T = 110 K) (Fig. 4a,b). In the calculated band structure (Fig. 4a), the electronic states between the Fermi level and 3 eV binding energy are mainly from the Iridium’s t_2*g*_ orbitals (white lines in Fig. 4a) while above 3 eV binding energy the contribution is mainly from the oxygen p orbitals (yellow lines in Fig. 4a). In addition, the orbital-resolved density of states (DOS) is also calculated where the peak position of *α*_0_ and *β*_0_ bands are well resolved (Fig. 4b). In the measured band structure, from the energy-second-derivative image (Fig. 4d), two bands are clearly observed that are marked as *α*_0_ and *β*_0_ between E_*F*_ and ~1 eV binding energy. These two bands show good agreement with the band structure calculations (Fig. 4a) and previous reports^7^,^8^,^9^,^10^,^11^,^12^,^13^,^14^. Also above 3 eV binding energy, the observed bands in the energy-second-derivative image (Fig. 4d) can find some good correspondence in the calculated band structure (Fig. 4a). The most dramatic difference between the measurements and calculations lies in the binding energy region above 1 eV. As seen in Fig. 4a, a couple of energy bands from Iridium are expected from the band structure calculations within the energy range of 1 ~ 3 eV but are not observed in the measured data (Fig. 4d). Instead, a number of nearly-vertical band features (*α*_1_, *α*_2_, *α*_3_ and *β*_1_ bands in Fig. 4e) appear within this energy range that are completely absent in the calculated band structure (Fig. 4a). The same is for the 3 ~ 6 eV binding energy range where some vertical bands are observed (Fig. 4e) but are not present in the calculated band structure at all (Fig. 4a).

Further inspection of the measured band structure indicates that the new nearly-vertical high energy bands appear to have a close connection with the lower energy *α*_0_ and *β*_0_ bands, as shown in Fig. 4c which summarizes all the observed bands on top of the original measured data. One can see that the three vertical bands *α*_1_, *α*_2_ and *α*_3_ merge into the *α*_0_ band at lower binding energy while the other vertical band *β*_1_ also merges into the lower binding energy *β*_0_ band. The low-energy electronic structure of Sr_2_IrO_4_ are mainly composed of t_2*g*_ bands that are split into two branches with the effective J_*eff*_ = 1/2 and J_*eff*_ = 3/2 because of the strong spin-orbit coupling^7^. It has been shown that the *β*_0_ band is predominantly with the J_*eff*_ = 1/2 character while the *α*_0_ band is mainly with the J_*eff*_ = 3/2 character (Fig. 4a,b)^7^. It is interesting to note that one vertical band (*β*_1_) emerges from the J_*eff*_ = 1/2 *β*_0_ band while three vertical bands (*α*_1_, *α*_2_ and *α*_3_) emerge from the J_*eff*_ = 3/2 *α*_0_ band (Fig. 4c), consistent with the orbital degeneracy of both the J_*eff*_ = 1/2 and 3/2 bands. These observations indicate the multi-orbital nature of the low energy electronic states in Sr_2_IrO_4_. In both the J_*eff*_ = 1/2 and 3/2 bands, we have observed such waterfall-like features splitting out of the original bands, this means that the high energy anomaly in Sr_2_IrO_4_ is a general feature appearing for all orbitals over a rather high energy scale.

To the best of our knowledge, such unusual high energy waterfall-like electronic structures are observed for the first time in Sr_2_IrO_4_. The appearance of nearly-vertical bands is quite unusual because it implies nearly infinite electron velocity if interpreted literally in the conventional band structure picture. This is reminiscent to the high energy waterfall feature observed in the high temperature cuprate superconductors^26^,^27^,^28^,^29^,^30^,^31^,^32^,^33^,^34^,^35^. The high energy behaviors are similar between the cuprates and Sr_2_IrO_4_ in a couple of aspects. First, the energy- and momentum-second-derivative images give different band structure^27^,^33^. For a conventional metal, the energy- and momentum-second-derivative images are supposed to produce similar band structure. The dichotomy between them already points to an exotic behavior and the effect of strong correlation. Second, nearly vertical bands are observed in the momentum-second-derivative images. The behavior in Sr_2_IrO_4_ is even more dramatic in that several bands show such waterfall-like high energy features (Fig. 4e) while only one band in cuprates shows such a behavior^26^,^27^,^28^,^29^,^30^,^31^,^32^,^33^,^34^,^35^. Moreover, the high energy features in Sr_2_IrO_4_ extend over a much larger energy range (1 ~ 3 eV for Ir states) (Fig. 4) while it is in the scale of 0.4 ~ 1 eV in cuprates^26^,^27^,^28^,^29^,^30^,^31^,^32^,^33^,^34^,^35^. The high energy behavior in Sr_2_IrO_4_ is even more complicated, such as the observation of a vase-like shape near the Γ point (*α*_1_ band Fig. 2a,b).

The revelation of the high energy waterfall-like bands in Sr_2_IrO_4_ provides another system that can be used to compare and contrast with the cuprates in order to understand the origin of the high energy anomaly. In the cuprate superconductors, the high energy anomalous band has attracted extensive experimental^26^,^27^,^28^,^29^,^30^,^31^,^32^,^33^,^34^,^35^ and theoretical interest^36^,^37^,^38^,^39^,^40^,^41^,^42^,^43^,^44^,^45^,^46^,^47^,^48^,^49^,^50^,^51^,^52^,^53^ although there has been no consensus reached on its origin. The prime candidate for the anomalous high energy behavior can be simply an intrinsic property of a strong electron correlation system or Mott physics^30^,^36^,^38^,^44^,^45^,^46^,^48^,^49^,^51^,^52^. The second possibility is due to quasiparticle scattering with some electronic or bosonic excitations, such as phonons^28^, plasmons^39^, paramagnons^29^,^40^,^42^,^49^, and other spin and charge excitations^53^. It can also be due to other novel effects such as the spin-charge separation^27^, spin polarons^37^, photoemission matrix element effect^32^, charge modulations^41^, quantum critical fluctuation^43^, in-gap band-tails^47^ and so on. Compared with the cuprates where there is a strong electron correlation (5–7 eV)^2^,^19^,^54^, the electron correlation in *5d* transition metal oxide Sr_2_IrO_4_ is much weaker (1 ~ 3 eV) owing to the much extended *5d* orbitals^7^,^19^. On the other hand, due to heavier atomic mass, the spin-orbit coupling becomes an order of magnitude stronger(~0.4 eV)^4^,^7^,^19^,^55^,^56^ in the *5d* transition metal oxides than that in their *3d* counterparts (~20 meV), reaching a comparable energy scale with the on-site Coulomb repulsion U and the bandwidth W^7^,^55^. This indicates that the spin-orbit coupling provides a novel tuning parameter in dictating the ground state and physical properties of the *5d* transition metal oxides. While the strong electron correlation plays an important role in producing high energy anomaly in the cuprate superconductors, the observation of the high energy anomaly in Sr_2_IrO_4_ provides a new scenario where the high energy anomaly can be observed in a system with a moderate or weak electron correlation and strong spin-orbit coupling.

One further question comes to whether the moderate electron correlation or the strong spin-orbit coupling alone can produce such a high energy anomaly in Sr_2_IrO_4_ or it is a combined effect. It would be surprising if a moderate electron correlation alone in Sr_2_IrO_4_ can cause the high energy anomaly over much larger energy scale than that in cuprates which has much stronger electron correlation although the possibility cannot be fully ruled out. There is no observation of high energy anomaly reported in systems with dominant spin-orbit coupling like simple metal Bi^57^ or topological insulators^58^,^59^. The anomalous high energy features can be most likely a combined effect of both the electron correlation and the spin-orbit coupling. This is consistent with the proposition that, in order to understand the insulating behavior of Sr_2_IrO_4_, both the on-site Coulomb interaction and strong spin-orbit coupling are necessary^7^. It is also consistent with the recent observation of a high energy anomaly in UCoGa_5_ that exhibits a moderate electron correlation and strong spin-orbital coupling^60^. Exotic quasiparticles like a composite particle has been reported lately in Sr_2_IrO_4_^61^. How the combination of the moderate electron correlation and the strong spin-orbit coupling can lead to such anomalous high energy excitations in Sr_2_IrO_4_ needs further theoretical and experimental investigations.

Interestingly, Sr_2_IrO_4_ exhibits a number of features that are similar to those of the high temperature cuprate superconductors. First, its crystal structure^5^ is similar to that of a parent compound La_2_CuO_4_^2^ with a slight distortion. Second, its insulating nature can be described by a J_*eff*_ = 1/2 Mott insulator model^7^ that is similar to the Mott insulator model for the parent compounds of the cuprate superconductors^2^. Third, the electron-doped Sr_2_IrO_4_ shows a single hole-like Fermi surface and Fermi arc^62^ that are quite reminiscent to that found in doped cuprate superconductors^25^. It is suggested that the half-filled doublet J_*eff*_ = 1/2 band would be mainly responsible for the low energy insulating physics in Sr_2_IrO_4_ as the role of the half-filled d_*x*2−*y*2_ band in cuprate parent compounds. The structural, electronic and magnetic similarities between Sr_2_IrO_4_ and the cuprate parent compound La_2_CuO_4_^23^ imply potential realization of superconductivity in doped Sr_2_IrO_4_^24^. Our present observation of anomalous high energy waterfall-like feature in Sr_2_IrO_4_ adds one more prominent similarity to that in the cuprate superconductors.

In summary, our ARPES measurements over a wide energy window have revealed for the first time a new phenomenon of the high energy anomalous bands in Sr_2_IrO_4_. It resembles the high energy waterfall feature observed in high temperature cuprate superconductors. While the low energy electron excitations in Sr_2_IrO_4_ can be described properly by considering both the on-site Coulomb repulsion and the strong spin-orbit coupling^7^, the high energy anomalous bands cannot be understood in the framework of the existing band structure calculations. Different from the cuprate superconductors where strong electron correlation plays an important role in producing high energy anomalies, the present results in Sr_2_IrO_4_ provides a new scenario that high energy anomaly can occur in a system with moderate or weak electron correlation and strong spin-orbit coupling. We hope these experimental observations can stimulate further theoretical work in understanding the anomalous electronic behaviors in Sr_2_IrO_4_ in particular, and the high energy anomaly in other materials in general.

## Methods

The Sr_2_IrO_4_ single crystals were synthesized by flux method^6^. High-resolution angle-resolved photoemission measurements were carried out on our lab system equipped with a Scienta R4000 electron energy analyzer^63^. We use helium discharge lamp as the light source that can provide photon energy of h*υ* = 21.218 eV (helium I). The overall energy resolution was set at 20 meV. The angular resolution is ~0.3 degree. The Fermi level is referenced by measuring on a clean polycrystalline gold that is electrically connected to the sample. The sample was cleaved *in situ* and measured at ~20 K in ultra-high vacuum with a base pressure better than 5 × 10^−11^ Torr. The measurements were carried out on different samples for several times and the results are reproducible.

## Additional Information

**How to cite this article**: Liu, Y. *et al*. Anomalous High-Energy Waterfall-Like Electronic Structure in 5*d* Transition Metal Oxide Sr_2_IrO_4_ with a Strong Spin-Orbit Coupling. *Sci. Rep*. **5**, 13036; doi: 10.1038/srep13036 (2015).

## Supplementary Material

Supplementary Information

## Figures and Tables

**Figure 1:**
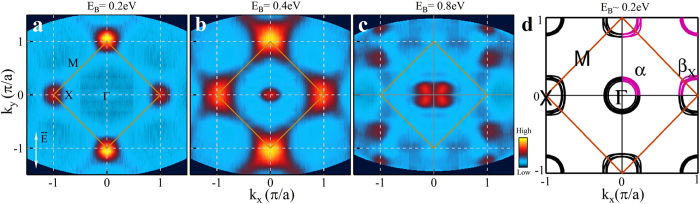
Measured constant energy contours of Sr_2_IrO_4_ and its comparison with calculations. (**a**–**c**) represent constant energy contours of the spectral weight distribution for Sr_2_IrO_4_ measured at ~20 K at different binding energies (E_*B*_) of 0.2 eV, 0.4 eV, and 0.8 eV, respectively. (**d**) is the calculated constant energy contour at a binding energy of ~0.2 eV by including on-site Coulomb repulsion and spin-orbit coupling^7^. The orange lines denote the antiferromagnetic Brillouin zone boundary for the IrO_2_ plane.

**Figure 2:**
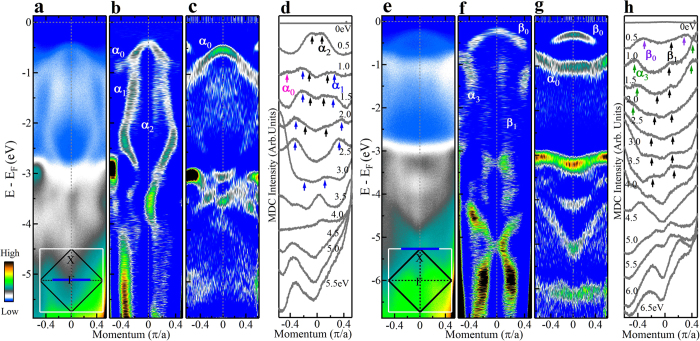
Typical band structures of Sr_2_IrO_4_ along high-symmetry cuts in a large energy range. (**a**) Original photoemission image of Sr_2_IrO_4_ measured along a high-symmetry cut across Γ; the location of the cut is shown as a solid blue line in the inset. (**b**,**c**) are corresponding momentum-second-derivative and energy-second-derivative images of (**a**), respectively. (**d**) Momentum distribution curves (MDCs) at different binding energies obtained from (**a**). (**e**) Original photoemission image measured along a high-symmetry cut across X; the location of the cut is shown as a solid blue line in the inset. (**f**,**g**) are corresponding momentum-second-derivative and energy-second-derivative images of (**e**), respectively. (**h**) MDCs at different binding energies obtained from (**e**).

**Figure 3:**
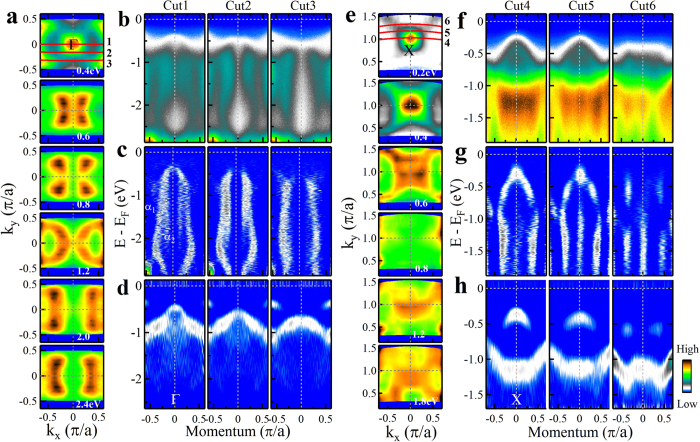
Momentum dependence of the band structures around Γ and X regions. (**a**) Constant energy contours around Γ point at different binding energies from 0.4 eV (top panel) to 0.6, 0.8, 1.2, 2.0 and 2.4 eV (bottom panel). (**b**) Original photoemission images measured along different momentum cuts around Γ. The location of the momentum cuts are shown as red lines in the top panel of (**a**). (**c**,**d**) are corresponding momentum-second-derivative and energy-second-derivative images of (**b**), respectively. (**e**) Constant energy contours around X point at different binding energies from 0.2 eV (top panel) to 0.4, 0.6, 0.8, 1.2 and 1.8 eV (bottom panel). (**f**) Original photoemission images measured along different momentum cuts around X. The location of the momentum cuts are shown as red lines in the top panel of (**e**). (**g**,**h**) are corresponding momentum-second-derivative and energy-second-derivative images of (**f**), respectively.

**Figure 4:**
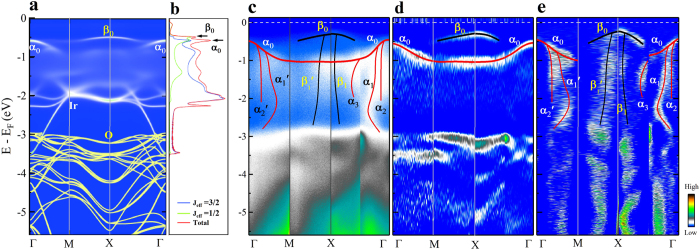
Calculated and measured overall band structure of Sr_2_IrO_4_. (**a**) Band structure of Sr_2_IrO_4_ by DMFT calculations along high symmetry line in the first Brillouin zone. The white lines are the LDA + DMFT calculation on Iridium’s t_2*g*_ orbitals while the yellow lines are the LDA calculation on Oxygen p orbitals. (**b**) Calculated density-of-states for the J_*eff*_ = 3/2 and J_*eff*_ = 1/2 states, and the total density-of-states of the Iridium orbitals. (**c**) Overall measured original photoemission image of Sr_2_IrO_4_ along high-symmetry cuts. The observed bands are overlaid on top of the original data. (**d**,**e**) are corresponding energy-second-derivative and momentum-second-derivative images of (**c**), respectively. The black and red lines are guides to the eye for the bands that can be resolved. *α*_1_′, *α*_2_′ and *β*_1_′ bands are equivalent bands to the *α*_1_, *α*_2_, and *β*_1_ bands along other symmetry cuts.
