# Supplementary material for: Anomalous High-Energy Waterfall-Like Electronic Structure in 5 d Transition Metal Oxide Sr2IrO4 with a Strong Spin-Orbit Coupling
Source: Sci Rep. 2015 Aug 12;5:13036. doi: 10.1038/srep13036 (PMC4533319; doi:10.1038/srep13036)
Supplement: Supplementary Information [file srep13036-s1.pdf]

Supporting Materials for

Anomalous High-Energy Waterfall-Like Electronic Structure in 5d Transition Metal Oxide Sr_2IrO_4 with a Strong Spin-Orbit Coupling

Yan Liu[1], Li Yu[1,*], Xiaowen Jia[1], Jianzhou Zhao[1], Hongming Weng[1], Yingying Peng[1], Chaoyu Chen[1], Zhuojin Xie[1], Daixiang Mou[1], Junfeng He[1], Xu Liu[1], Ya Feng[1], Hemian Yi[1], Lin Zhao[1], Guodong Liu[1], Shaolong He[1], Xiaoli Dong[1], Jun Zhang[1], Zuyan Xu[3], Chuangtian Chen[3], Gang Cao[4], Xi Dai[1], Zhong Fang[1] and X. J. Zhou[1,2,*]

[1]*Beijing National Laboratory for Condensed Matter Physics, Institute of Physics, Chinese Academy of Sciences, Beijing 100190, China*

[2]*Collaborative Innovation Center of Quantum Matter, Beijing, China*

[3]*Technical Institute of Physics and Chemistry, Chinese Academy of Sciences, Beijing 100190, China*

[4]*Department of Physics and Astronomy, University of Kentucky, Lexington, KY 40506*

(Dated: April 2, 2015)

1. Band structures and corresponding photoemission spectra of Sr_2IrO_4 along several typical momentum cuts.

The band structures and corresponding photoemission spectra along several typical momentum cuts are shown in Figs. S1-S3 in a wide energy window. Fig. S1 shows the band structure and corresponding photoemission spectra (Energy Distribution Curves, EDCs) near Γ point (Fig. S1a) and X point (Fig. S1c) and their corresponding EDCs. For these two cuts, the high energy band structure anomaly has been discussed in the main text (Fig. 2). Figs. S2 and S3 show original data, second derivatives of the original data with respect to momentum and energy, and the corresponding photoemission spectra for other two high-symmetry momentum cuts: one along the Γ-M direction (Fig. S2) and the other along the M-X direction. In both cases, the high energy anomalous band structures can be clearly observed in the MDC-second derivative images (Fig. 2b and Fig. 3b).

2. MDC and EDC fitting of the Sr_2IrO_4 band structures near the Γ point.

In order to resolve the low energy band structures of Sr_2IrO_4 near the Γ point more clearly, we have taken high-resolution ARPES measurements and carried out MDC- and EDC-fitting of the observed bands. Both the MDC and EDC fittings are done by using the Lorentzian lineshape. The EDC-fitted bands, α_0 (black line in Fig. 4a) and β_0 (pink line in Fig. 4a), are consistent with the bands shown in the EDC-second-derivative image (Fig. 4b). Two nearly vertical bands can be clearly resolved and be MDC-fitted into α_1 and α_2 bands (red lines) in Fig. 4a. These MDC-fitted bands are also consistent with the bands shown in the MDC-second-derivative image (Fig. 4c).

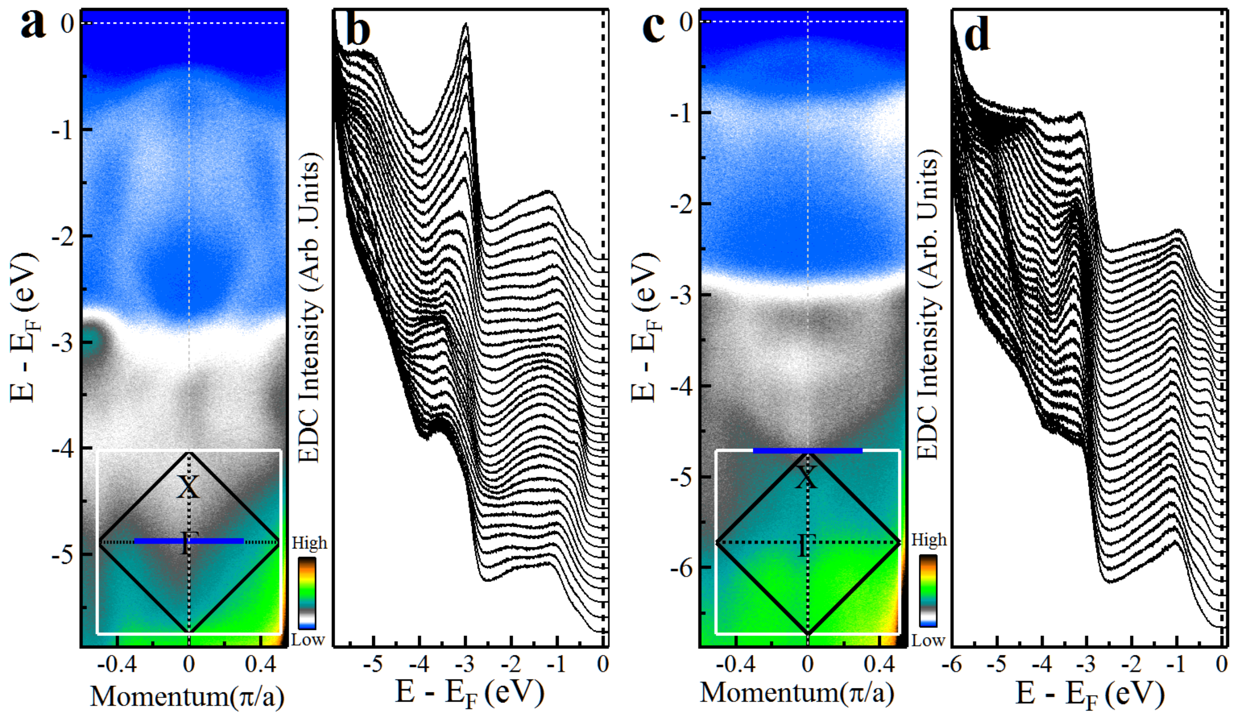

FIG. 1: **Fig. S1: Band structure and photoemission spectra of Sr_2IrO_4 across the Γ and X points.** (**a**). Band structure along the momentum cut across the Γ point, as marked by the blue line in the bottom inset. Its corresponding photoemission spectra (Energy Distribution Curces, EDCs) are shown in (**b**). (**c**). Band structure along the momentum cut across the X point, as marked by the blue line in the bottom inset. Its corresponding photoemission spectra (EDCs) are shown in (**d**).

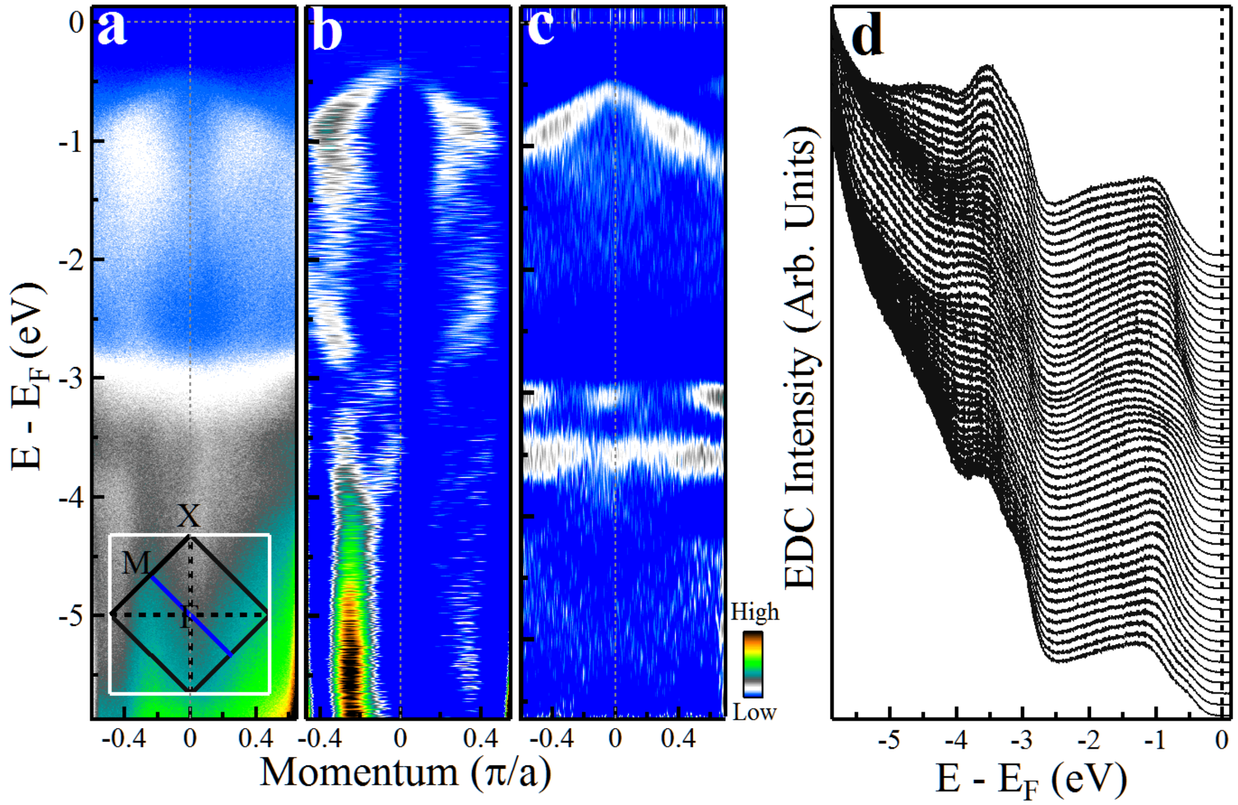

FIG. 2: **Fig. S2: Band structure and photoemission spectra of Sr_2IrO_4 along the Γ-M direction.** (**a**). Band structure along the Γ-M direction across the Γ point, as marked by the blue line in the bottom inset. Its corresponding second derivative images with respect to momentum and energy are shown in (**b**) and (**c**) respectively. (**d**). The corresponding photoemission spectra (EDCs) of (**a**).

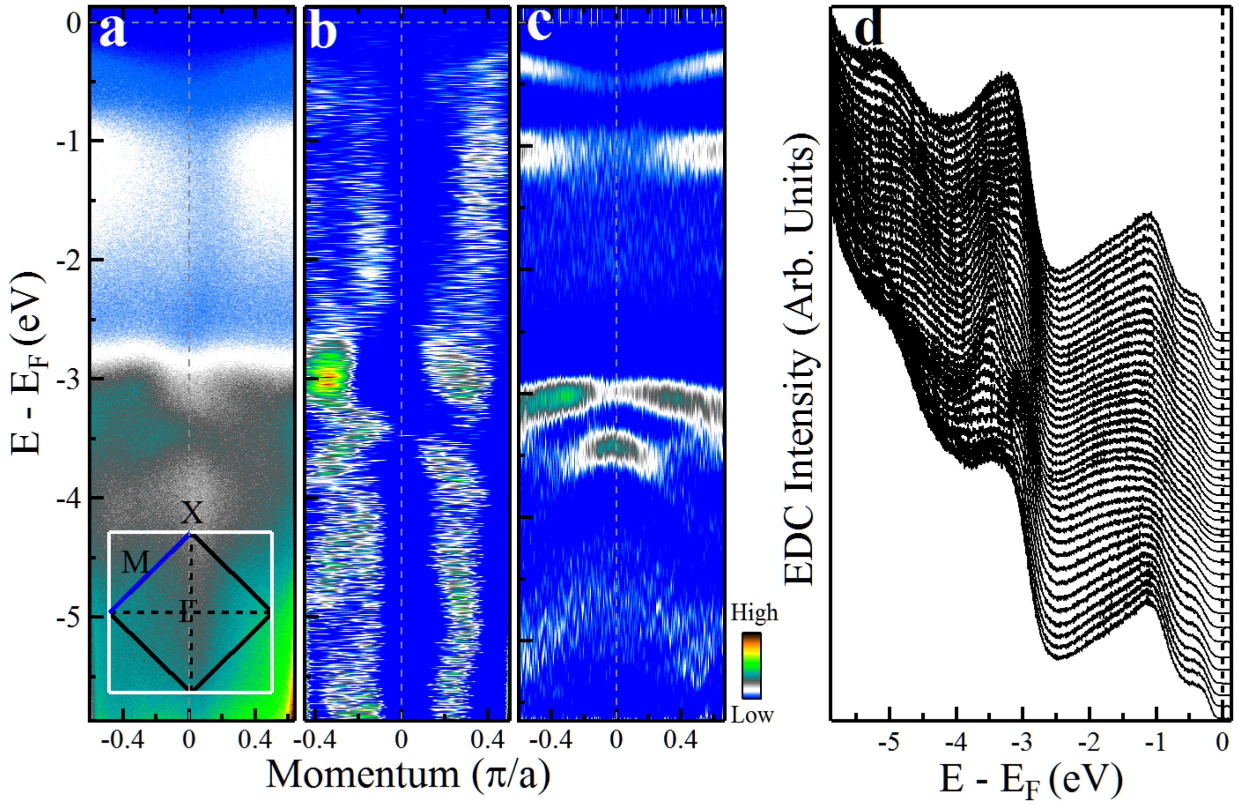

FIG. 3: **Fig. S3: Band structure and photoemission spectra of Sr_2IrO_4 along the M-X direction.** (**a**). Band structure along along the M-X direction across the M point, as marked by a blue line in the bottom inset. Its corresponding second derivative images with respect to momentum and energy are shown in (**b**) and (**c**), respectively. (**d**) shows the corresponding photoemission spectra (EDCs).

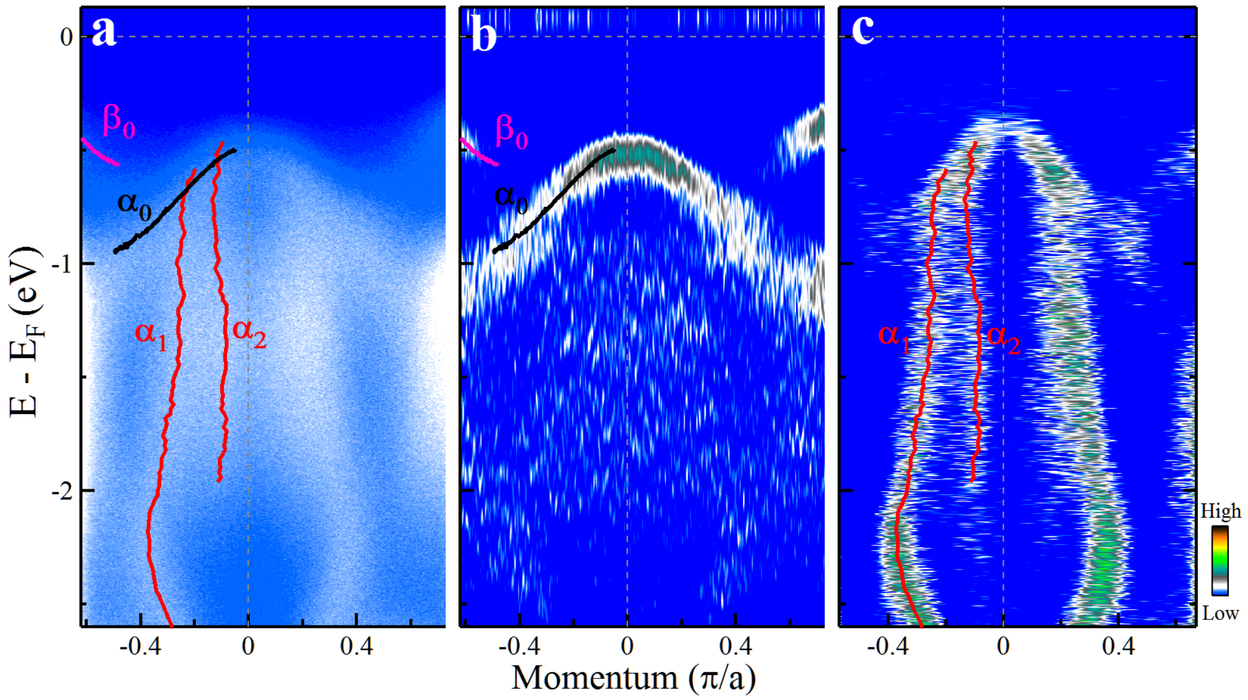

FIG. 4: **Fig. S4: MDC and EDC fit of the measured Sr_2IrO_4 band structures around the Γ point.** (**a**). Band structure measured along the ΓX direction centered at Γ point. The red lines are the MDC fit of the α_1 and α_2 band, and the black line is the EDC fit of the α_0 band. The purple line is the EDC fit of the β_0 band. The corresponding EDC and MDC second derivative image are shown in (**b**) and (**c**), respectively, where the EDC and MDC fitted lines are marked.
